# Human ADSC xenograft through IL-6 secretion activates M2 macrophages responsible for the repair of damaged muscle tissue

**DOI:** 10.1186/s13287-019-1188-y

**Published:** 2019-03-13

**Authors:** Ewelina Pilny, Ryszard Smolarczyk, Magdalena Jarosz-Biej, Alina Hadyk, Agnieszka Skorupa, Mateusz Ciszek, Łukasz Krakowczyk, Natalia Kułach, Danuta Gillner, Maria Sokół, Stanisław Szala, Tomasz Cichoń

**Affiliations:** 1 Center for Translational Research and Molecular Biology of Cancer, Maria Sklodowska-Curie Institute - Oncology Center, Gliwice Branch, Wybrzeże Armii Krajowej 15 Street, 44-101 Gliwice, Poland; 2 Department of Organic Chemistry, Biochemistry and Biotechnology, Silesian University of Technology, Księdza Marcina Strzody 9 Street, 44-100 Gliwice, Poland; 3 Department of Medical Physics Maria Sklodowska-Curie Institute -Oncology Center, Gliwice Branch, Wybrzeże Armii Krajowej 15 Street, 44-101 Gliwice, Poland; 4 Department of Oncologic and Reconstructive Surgery, Maria Sklodowska-Curie Institute -Oncology Center, Wybrzeże Armii Krajowej 15 Street, 44-101 Gliwice Branch, Gliwice, Poland; 5 Department of Animal Physiology and Ecotoxicology, Faculty of Biology and Environmental Protection, University of Silesia, Bankowa 12 Street, 40-007 Katowice, Poland

**Keywords:** hADSCs, IL-6, M2 macrophages, Blood vessels, Repairing damaged muscle, Xenograft

## Abstract

**Background:**

Adipose-derived mesenchymal stromal cells (ADSCs) are multipotent stromal cells. The cells secrete a number of cytokines and growth factors and show immunoregulatory and proangiogenic properties. Their properties may be used to repair damaged tissues. The aim of our work is to explain the muscle damage repair mechanism with the utilization of the human adipose-derived mesenchymal stromal cells (hADSCs).

**Methods:**

For the hADSCs isolation, we used the subcutaneous adipose tissue collected during the surgery. The murine hind limb ischemia was used as a model. The unilateral femoral artery ligation was performed on 10–12-week-old male C57BL/6NCrl and NOD SCID mice. The mice received PBS^−^ (controls) or 1 × 10^6^ hADSCs. One, 3, 7, 14 and 21 days after the surgery, we collected the gastrocnemius muscles for the immunohistochemical analysis. The results were analyzed with relevant tests using the Statistica software.

**Results:**

The retention time of hADSCs in the limb lasted about 14 days. In the mice receiving hADSCs, the improvement in the functionality of the damaged limb occurred faster than in the control mice. More new blood vessels were formed in the limbs of the mice receiving hADSCs than in limbs of the control mice. hADSCs also increased the infiltration of the macrophages with the M2 phenotype (7-AAD^−^/CD45^+^/F4/80^+^/CD206^+^) into the ischemic limbs. hADSCs introduced into the limb of mice secreted interleukin-6. This cytokine stimulates the emergence of the proangiogenic M2 macrophages, involved, among others, in the repair of a damaged tissue. Both macrophage depletion and IL-6 blockage suppressed the therapeutic effect of hADSCs. In the mice treated with hADSCs and liposomes with clodronate (macrophages depletion), the number of capillaries formed was lower than in the mice treated with hADSCs alone. Administration of hADSCs to the mice that received siltuximab (human IL-6 blocker) did not cause an influx of the M2 macrophages, and the number of capillaries formed was at the level of the control group, as in contrast to the mice that received only the hADSCs.

**Conclusions:**

The proposed mechanism for the repair of the damaged muscle using hADSCs is based on the activity of IL-6. In our opinion, the cytokine, secreted by the hADSCs, stimulates the M2 macrophages responsible for repairing damaged muscle and forming new blood vessels.

## Background

The critical limb ischemia is an advanced stage of a peripheral artery disease in which stenosis and occlusion of the arteries occur. One of the methods of treatment for this disease is therapeutic angiogenesis. The therapy utilizes growth factors or cells that secrete factors which stimulate the formation of new blood vessels [1–3]. An increased number of blood vessels in the ischemic limb model was observed after the administration of mesenchymal stromal cells isolated from the adipose tissue (ADSC) [4].

ADSCs are multipotent stromal cells which secrete a number of cytokines and growth factors and show immunoregulatory and proangiogenic properties. A dominant cytokine secreted by ADSC is interleukin 6 (IL-6) [5], which is a pleiotropic cytokine. This cytokine is involved in the immune response and influences the formation of new blood vessels by regulating the vascular endothelial growth factor (VEGF) [6]. IL-6 is also one of the cytokines participating in the process of the polarization of the proinflammatory M1 macrophages into the immunosuppressive, proangiogenic M2 macrophages [7, 8]. M2 macrophages are involved in the inhibition of the inflammatory response, as in, they initiate the tissue repair processes. They secrete and release many proangiogenic factors (chemokines, cytokines, growth factors) that stimulate endothelial cells to migrate and proliferate [9].

Our results indicate that human adipose-derived mesenchymal stromal cells (hADSCs) introduced into the ischemic limb secrete interleukin 6. In our opinion, the cytokine activates M2 macrophages responsible for the formation of new blood vessels and eventually for the repair of the damaged tissues.

## Methods

### Ethical statement

The experiments with animals were performed in accordance with the Declaration of Helsinki and with the approval of the Local Ethics Committee for Animal Experiments in Katowice (Permit Number: KB430-17/14).

### Animals

Mice (8 to 10-week-old, C57Bl/6NCrl and NOD SCID, males) were obtained from Charles River Breeding Laboratories. All mice were housed in the Maria Sklodowska-Curie Institute-Oncology Center, Gliwice Branch (Poland) in a HEPA-filtered Allentown’s IVC System (Allentown Caging Equipment Co, NJ, USA). Mice (n5) were kept in cages with an area of 435 cm2 and a height of 13.3 cm (Allentown Caging Equipment Co).

The cage bed was a dust-free, resin-free, autoclavable litter of aspen wood (MAXIÐLTE 004, ABEDD Vertriebs GmbH, Wien, OÈ sterreich). The environment was enriched with nesting materials of aspen wood fibers with 2.5 mm (NBF E-011, Allentown Caging Equipment Co). Mice were kept under 12-h dark/12-h light cycle in SPF animal facility. The relative humidity in the air-conditioned rooms was maintained at 50 ± 55% and temperature at 21 ± 22ÊC. The animals received a total pathogen-free standard diet (Altromin 1314, Altromin Spezialfutter GmbH & Co. KG, Germany) and water ad libitum throughout the whole study. This study was carried out in strict accordance with the recommendations in the Guide for the Care and Use of Laboratory Animals of the National Institutes of Health. All experiments on animals are conducted in accordance with the 3R rule and at the same time with taking into consideration the right number of the animals in groups making it possible to run the statistical analysis.

### The isolation of hADSCs

The subcutaneous adipose tissue specimens (*n* = 12) were collected during planned surgery in Maria Skłodowska-Curie Memorial Cancer Center and Institute of Oncology, Gliwice Branch (Poland). Researches using human adipose tissue has been accepted by the Committee on the Bioethics Maria Sklodowska-Curie Institute - Oncology Center, Gliwice Branch (Permit Number: KB/430-35/14).

The adipose tissue was rinsed with PBS^−^ (Gibco BRL, Paisley, UK) and supplemented with 1% of FBS (Gibco). The tissue was incubated with NB4 collagenase solution (0.4 U/mL, Serva Electrophoresis, Heidelberg, Germany) in Ham’s F-12 medium (Life Technologies, Carlsbad, CA, USA) supplemented with 10% of FBS and Ca^2+^ (2.5 mM, Sigma Aldrich, St Louis, MO,USA) for 70 min. in 37 °C with shaking (250 rpm). After the digestion, the cell suspension was centrifuged (1500 rpm, 10 min) and the cells were suspended in red blood cell lysis buffer (Miltenyi Biotec, Bergisch Gladbaels, Germany) for 10 min at RT. After the centrifugation, the cells were suspended in PBS^−^ and filtered through 70-μm cell strainer. The suspension of single cells was placed on plastic culture dishes (6 × 10^6^ cells/10 cm plate) in DMEM medium with high glucose concentration (4.5 g/L) (Life Technologies) supplemented with 20% FBS and antibiotics (penicillin and streptomycin, Sigma Aldrich) (acc. to [10]). Cells were incubated at 37 °C with 5% of CO_2_. After 72 h, the cells were washed twice with PBS^−^ to remove nonadherent cells and a fresh culture medium was added to the plates. The medium was changed every 2–3 days. The experiments were conducted on cells after the second passage.

### Cell culture

Mouse adipose-derived (mADSCs) and mouse bone marrow-derived (mBM-MSCs) mesenchymal stromal cells were used. Murine (L929) and human (NHDF, GM07492) fibroblasts were also used. The mADSCs were isolated from the inguinal adipose tissue of 8–10-week-old C57BL/6NCrl female mice. The tissue was collected and incubated with NB4 collagenase solution (0.4 U/mL, Serva Electrophoresis) in Ham’s F-12 medium (Life Technologies) supplemented with 10% FBS and Ca^2+^ (2.5 mM, Sigma Aldrich) for 70 min at 37 °C with shaking (250 rpm). After the filtration through a 70-μm cell strainer and centrifugation, the cells were suspended in a medium and placed on culture dishes. mBM-MSCs were isolated from 6–8-week-old C57BL/6NCrl female mice. Femurs and tibias from mice were dissected and rinsed with IMDM medium (Gibco). The bone fragments were incubated with NB4 collagenase solution (3 mg/mL) in IMDM medium with 10% FBS and Ca^2+^ (2.5 mM) (250 rpm with shaking, 37 °C, 3 h). After the filtration through a 70-μm cell strainer and centrifugation, the cells were suspended in a medium and placed on culture dishes. L929 cells (NCTC clone 929, ATCC, Manassas, USA) were cultured in RPMI 1640 medium (Life Technologies) supplemented with 10% of heat-inactivated FBS and antibiotics (penicillin and streptomycin, Sigma Aldrich). NHDF (Lonza, Basel, Switzerland) and GM07492 (Coriell Cell Repositories, NJ, USA) cells were cultured in DMEM medium with high glucose and supplemented with 10% FBS and antibiotics (penicillin and streptomycin). mADSCs and mBM-MSC were cultured in IMDM medium supplemented with 10% FBS and antibiotics (penicillin and streptomycin). Cells were incubated at 37 °C with 5% of CO_2_.

### Analysis of the phenotype and differentiation capacity of hADSCs

The single-cell suspension was incubated for 30 min at 4 °C with antibodies directed against the following human antigens: CD105-APC, CD29-FITC, CD73-PE, CD90-PECy7, CD44-FITC, CD34-PE-Cy7, CD31-FITC, KDR-PE, CD146-PE, CD45-PE, Lin-FITC, HLA-DR-PECy7 (BD Pharmingen, San Diego, CA, USA), and suitable isotype control antibodies. 7-AAD (7-amino-actinomycin D; eBioscences) was used to stain nonviable cells just before running the flow analysis. The samples were analyzed using a flow cytometer (BD FACSCanto™, BD Biosciences). To examine the adipogenic and osteogenic differentiation capacity of the isolated cells, we used The Human Mesenchymal Stem Cell Functional Identification Kit (RayBiotech, Norcross, GA, USA) in accordance with the manufacturer’s instructions. The hADSCs were cultured for 21 days in the adipogenic and osteogenic differentiation medium. The ability of the hADSCs to differentiate into osteoblasts was assessed by histochemical staining using Alizarin Red (Aldrich). The cells were visualized using an Eclipse 80i microscope (Nikon Instruments Inc., Melville, NY, USA). The ability of hADSCs to differentiate into adipocytes was assessed by immunofluorescence staining using an antibody directed against the FABP4 (Fatty Acid Binding Protein, Abcam) and a secondary antibody linked to an FITC fluorochrome (Vector Laboratories). Fluorescence imaging of the stained cells was performed using a LSM710 confocal microscope (Carl Zeiss Microscopy GmbH).

### Determination of IL-6 secreted in vitro by hADSCs

hADSCs were cultured for 24 h in standard conditions. Then, the cells were fixed with 4% paraformaldehyde (Sigma Aldrich) and stained with Phalloidin conjugated with Alexa Fluor 594 (Abcam, Cambridge, UK). The sections were incubated with an antibody directed against human interleukin 6 (Abcam). Subsequently, the sections were incubated with a secondary antibody conjugated with FITC (Vector Laboratories, Burlingame, CA, USA). Sections were mounted in VECTASHIELD Mounting Medium with DAPI (Vector Laboratories). Imaging of the fluorescence of the stained sections was performed with the confocal microscope LSM710 (Carl Zeiss Microscopy).

### Determination of IL-6 secreted by hADSCs and mADSCs using ELISA test

hADSCs and mADSCs were cultured in standard conditions in serum-containing complete medium. On the next day of the culture, the medium was replaced with DMEM without the serum. After 4 h, the medium was replaced with a fresh one. After 2, 4, 8, 16, 24, and 48 h, the medium was collected and stored at − 80 °C until needed. ELISA kits (eBioscience) were used according to the manufacturer’s instructions to determine the levels of the secreted IL-6.

The total protein amount in the conditioned medium was determined by the Bradford method.

### The murine model of hind limb ischemia

The unilateral femoral artery ligation was performed on 10–12-week-old male C57BL/6NCrl (immunocompetent) and NOD SCID (immunodeficient) mice. The mice were originally purchased from Charles River Laboratories (Wilmington, MA, USA). Animals were anesthetized with 2% isoflurane (MiniVent Model 845, Harvard Apparatus, USA). The skin from the inner side of the left hind limb was incised over a length of about 1 cm, and the superficial femoral artery was exposed. The artery was ligated at two points using surgical sutures. First ligation was performed about 0 .5cm from the hip joint and the other about 1 cm further down from the first ligation. The skin incisions were closed. The body temperature was maintained with heating pads until the animals recovered from surgery. An hour after the ligation, the administrations of hADSCs, mADSCs, mBM-MSCs, L929, GM07492, and NHDF cells (1 × 10^6^ cells suspended in 100 μL of PBS-) into the gastrocnemius muscles were performed. We also administrated 150 ng of recombinant murine or human IL-6 (Abcam) in a total volume of 100 μL in PBS into the gastrocnemius muscle once an hour after the ligation. The control mice were injected with 100 μL of PBS^−^.

### Angiography

The magnetic resonance angiography experiments were performed on a 9.4 T vertical 89-mm-bore system (Bruker BioSpin, Rheinstetten, Germany) equipped with a Bruker Micro 2.5 gradient system and a transmit/receive birdcage radio frequency coil with an inner diameter of 30 mm. During data collection, animals were anesthetized with 2–3% solution of sevoflurane. Body temperature and respiration rate were monitored using ECG/respiratory unit (SA Instruments, Inc., Stony Brook, NY, USA). Images of vascular system were acquired using a multi-slice 2D TOF (time of flight) flow compensated sequence using the following parameters: TE = 3.1 ms, TR = 12 ms, flip angle = 80°, field of view 2 × 3cm^2^, matrix size 200 × 300, slice thickness 0.3 mm, inter-slice distance 0.2 mm, and number of signal averages 3.

### In vivo assessment of limb function

A semiquantitative assessment of the impaired use of the ischemic limb was performed using the following criterion: 3—dragging of foot, 2—no dragging but no plantar flexion, 1—plantar flexion, and 0—flexing the toes to resist the gentle traction on the tail.

### Flow cytometric analysis of macrophages in the ischemic muscle

Flow cytometry was used to determine the subsets of macrophages in the ischemic muscles. Seven days after the artery ligation and hADSCs, mADSCs, or PBS^−^ administrations, the muscles were collected, minced, and digested with collagenase II solution for 1 h at 37 °C (500 U/mL; Gibco). The red blood cells were lysed using 0.15 M ammonium chloride solution (Sigma Aldrich). The cell suspensions were filtered through 70-μm cell strainer. The dead cells were removed by centrifugation using Histopaque-1077 gradient (Sigma Aldrich). Cells were incubated with rat anti-mouse CD16/CD32 blocking antibody (eBioscience) and then incubated with anti-CD45 (BioLegend), anti-F4/80 (eBioscience), anti-CD206 (Bio-Rad), and anti-CD86 (Biologend) antibodies for 30 min. All FACS-analyzed populations were gated in a 7-AAD (eBioscience) window to enrich for live cells. To analyze the macrophage phenotype, 7-AAD-viable muscle derived cells and CD45 (to identify lymphoid cells) were gated and then F4/80 (to identify macrophages) and CD206 (to identify CD45^+^/ F4/80^+^/ CD206^+^; M2 macrophages) and CD86 (to identify CD45^+^/ F4/80^+^/ CD86^+^, M1 macrophages). Gates dividing negative from positive cells were based on the isotype antibody control probes in the flow cytometric analysis (BD FACSCanto, BD) [23].

### Determination of human and murine interleukin-6 in serum and tissue after ADSCs injection

Seven days after the artery ligation and hADSCs, mADSCs, or PBS^−^ administration, mice were sacrificed by cervical dislocation. The blood was collected into BD Vacutainers and left for 30 min for clotting and then centrifuged. The serums were collected and stored at − 80 °C until needed. The gastrocnemius muscles were collected and stored at − 80 °C until needed. The frozen tissues were homogenized and incubated in cell lysis buffer with proteases cocktail (Thermo Scientific). The homogenates were centrifuged at 4000 rpm for 10 min at 4 °C. The supernatants from homogenized muscles were collected. The concentration of human and murine IL-6 in collected supernatants and serum was determined by ELISA (eBioscience) in accordance with the manufacturer’s instruction. The total protein amount in the homogenates was determined by the Bradford method.

### The histochemical staining

One, 3, 7, 14, and 21 days after the surgery, mice were sacrificed by cervical dislocation and the gastrocnemius muscles were collected for immunohistochemical analysis. The collected muscles were frozen in liquid nitrogen and stored at − 80 °C until needed. Subsequently, frozen tissues were sectioned into 5-μm slices.

The frozen sections were examined histochemically (hematoxylin/eosin staining, Sigma Aldrich), and the analysis of the specimens was conducted using the Nikon Eclipse 80i microscope (Nikon Instruments Inc., Melville, NY, USA).

### The identification of hADSCs in gastrocnemius muscles

The hADSCs were identified in the gastrocnemius muscles using murine monoclonal primary antibody directed against the human LAMIN A + C—Nuclear Envelope Marker (Abcam, 1:100). Sections were stained in accordance with the manufacturer’s instruction for the Vector M.O.M. Fluorescein Kit (Vector Laboratories). To confirm IL-6 secretion in tested muscles, the sections were additionally incubated with an antibody against human interleukin 6 (Abcam). To confirm that macrophages were located at the site of hADSCs’ administration, the sections were additionally incubated with anti-CD206 antibody (Abcam). Subsequently, the sections were incubated with a Texas Red conjugated secondary antibody (Vector Laboratories). Sections were mounted in VECTASHIELD Mounting Medium with DAPI. Imaging of the fluorescence of the stained sections was performed with the confocal microscope LSM710 (Carl Zeiss Microscopy).

### The immunohistochemical assessment of blood vessels and macrophages in gastrocnemius muscles

To determine the newly formed vessels in gastrocnemius muscles after the hADSCs administration, the frozen sections were stained immunohistochemically. The frozen sections were incubated with antibodies directed against rat CD31 and rabbit CD146, α-SMA (Abcam). Additionally anti-F4/80 and anti-CD206 antibodies (Abcam) were used to identify M2 macrophages. Subsequently, sections were incubated with a secondary antibodies conjugated with Texas Red, FITC, or Alexa Fluor (Abcam). Sections were mounted in VECTASHIELD Mounting Medium with DAPI (Vector Laboratories). Imaging of the fluorescence of the stained sections was performed with the confocal microscope LSM710.

### The macrophages depletion

In order to deplete the macrophages, 200 μL of clodronate liposomes solution or PBS^−^ liposomes in an amount of 1 mg/mL (Clodronate Liposomes Organization, Vrije Universiteit, Netherlands) was injected i.v. using a 26-gauge needle. The liposomes were injected twice—48 h and 1 h—before the hADSCs or PBS^−^ administration to ensure depletion of resident macrophages and every 2/3 days after cell injection to deplete infiltrating macrophages. hADSCs or PBS^−^ were injected as previously described. On the 7th day of the experiment, the muscles were collected, frozen in liquid nitrogen, and stained to show mature macrophages (F4/80) and the M2 macrophages (CD206) to confirm the macrophages depletion. The effect of macrophages depletion on the formation of the new blood vessels was determined using anti-CD31 antibody staining as described previously.

### Blocking of human IL-6 in vivo

Blocking of human IL-6 secreted by hADSCs in vivo was achieved using an antibody against human IL-6—siltuximab (10 mg/kg) (*Janssen Cilag*, Turnhoutseweg, Belgium). Siltuximab was injected i.v. 2 days and 1 h prior and every 2/3 days after the hADSCs or PBS^**−**^ injection. hADSCs or PBS^**−**^ were administered as previously described. On the 7th day of the experiment, the muscles were collected, frozen in liquid nitrogen, and stained with anti-CD31 antibody as described previously. To confirm the effect of depletion of IL-6, secreted by hADSCs, on the macrophage infiltration, the muscles were stained with F4/80 and CD206 antibodies as previously described.

### Statistics

The results were analyzed with the appropriate tests using the Statistica software (Dell Inc. (2016). Dell Statistica (data analysis software system), version 13. software.dell.com). *P* values *<* 0.05 were considered statistically significant.

## Results

### The phenotype of hADSCs stabilized during cell culture

**Fig. 1:**
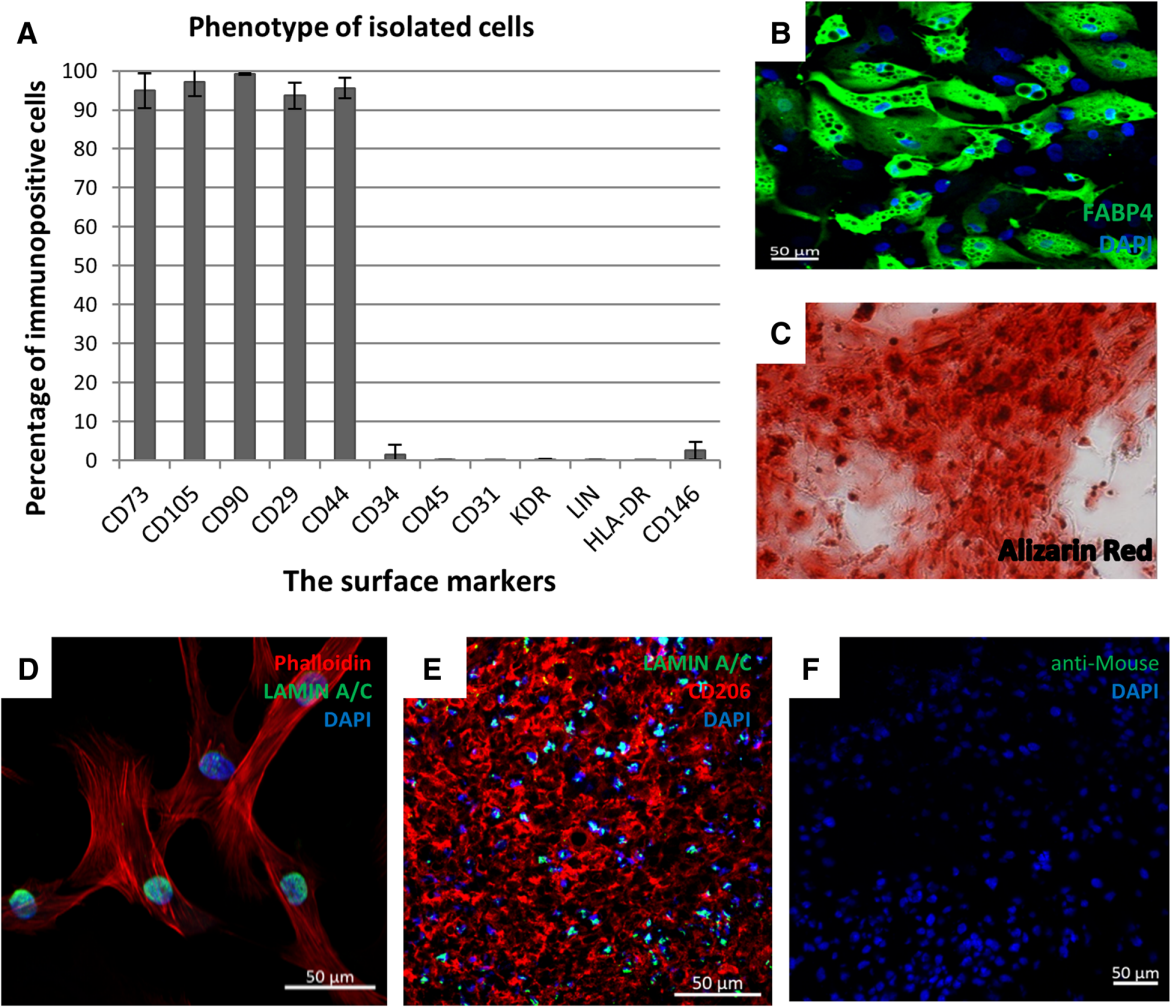
The hADSCs characteristics in vitro and their retention time after the administration to the murine muscle tissue. **a** Phenotype of hADSC (passage #2; *n* = 12 Flow cytometry). The phenotype fulfills all the accepted criteria for MSCs identification; **b** adipogenic differentiation—formation of lipid vesicles stained with FABP4 antibody (green, passage #2, *n* = 5); **c** osteogenic differentiation—calcium deposits stained with Alizarin Red (red, passage #2, *n* = 7, magn × 20); **d** immunofluorescence phalloidin staining of actin filaments (red) and Lamin A/C (green) in hADSCs in vitro. **e** The representative image of double immunofluorescent staining of human specific Lamin A/C (green) and macrophages (CD206, red) on day 7. Image shows that injected hADSC localize in clusters, presumably at the injection site. **f** Image shows control immunofluorescence staining with secondary Biotinylated Anti-Mouse and Fluorescein Avidin antibodies alone. Nuclei were stained with DAPI (blue), scale bars = 50 μm

The human hADSCs were isolated from subcutaneous adipose tissue from the abdominal region. Flow cytometry analysis showed that hADSCs expressed CD105, CD90, CD73, CD44, and CD29 surface markers and were negative for CD45, CD34, HLA-DR, KDR, and CD31 antigens (Fig. 1a). The phenotype of hADSCs was stabilized after the second passage during cell culture. Isolated cells in vitro differentiated towards adipocytes (Fig. 1b) and osteoblasts (Fig. 1c).

### The retention time of hADSCs cells in the injured limb is 14 days

On 3, 7, and 14 days after the hADSCs injection, we collected the gastrocnemius muscles and used immunohistochemical staining to identify the human nuclear protein—Lamin A/C in the murine muscles (Fig. 1e). It was observed that on day 14 after the surgery hADSCs were still present in the gastrocnemius muscles of the injured limbs (data not shown).

### The administration of the hADSCs cells to the injured limb accelerated its functional recovery

**Fig. 2:**
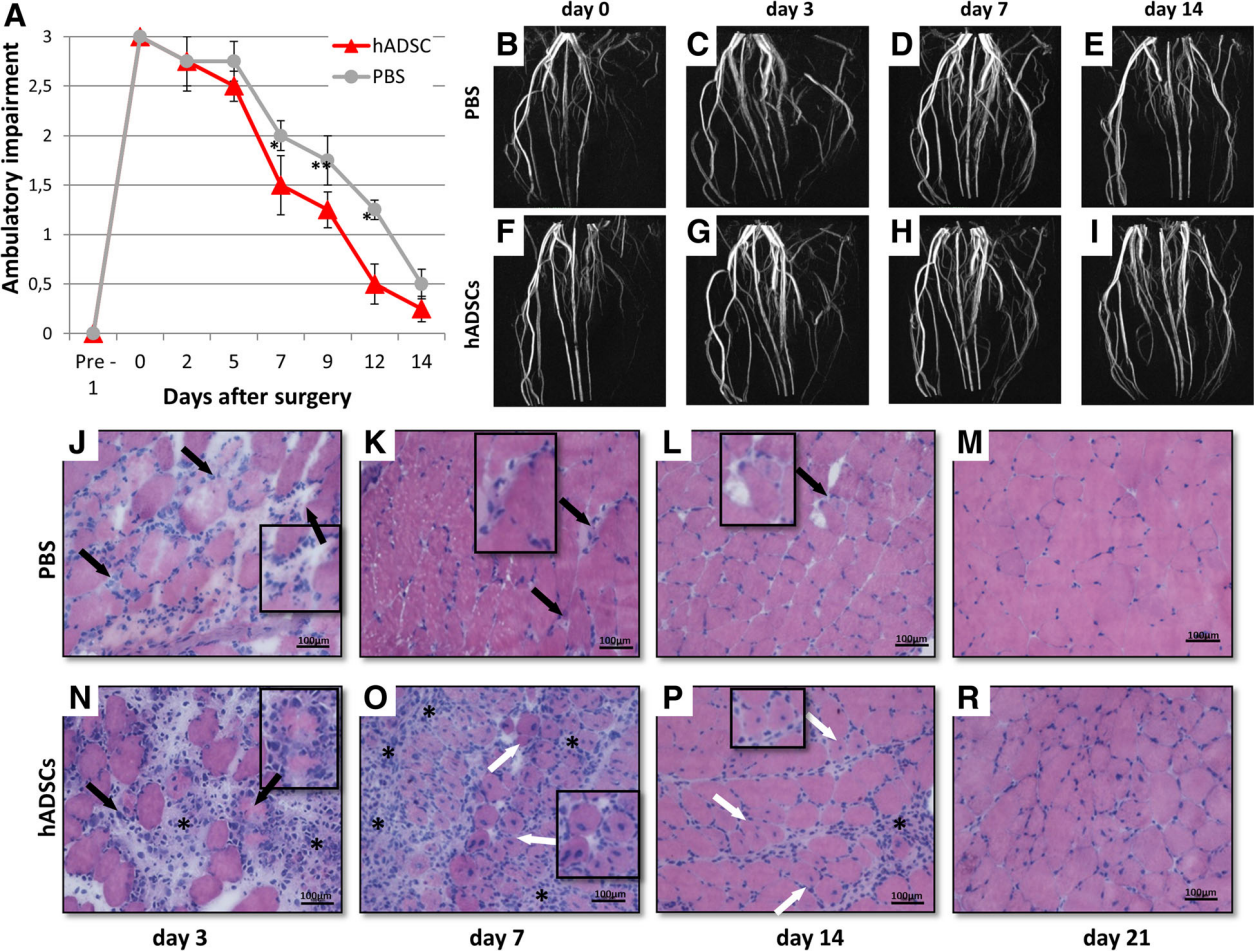
MR imaging of vascular system and muscle regeneration in mouse gastrocnemius muscle after the hindlimb ischemia. The mice given hADSC demonstrated improved functional outcomes as compared to the control mice (PBS^−^) (*n* = 10)*p < 0.05, **p < 0.01 by the *U* Mann-Whitney test (**a**). Effectiveness of femoral artery ligation and consecutive restoration of the blood flow in the ischemic hindlimbs were visually evaluated using magnetic resonance angiography. Representative magnetic resonance angiography images (maximum intensity projections) of mouse hindlimbs were acquired immediately after the femoral artery ligation (**b**—control mouse, **f**—hADSC treated mouse), on day 3 (**c**—control mouse, **g**—hADSC treated mouse), on day 7 (**d**—control mouse, **h**—hADSC-treated mouse) and on day 14 (**e**—control mouse, **i**—hADSC-treated mouse). The representative images of the transverse sections of the gastrocnemius muscle tissues by H&E staining after PBS^−^ (**j**, **k**, **l**, **m**) and hADSC (**n**, **o**, **p**, **r**) injection at 3, 7, 14, and 21 days after surgery. Necrotic muscle fibers (black arrows) with pale cytoplasm were observed at 3 (**j**), 7 (**k**), and 14 (**l**) days after the PBS^−^ administration. Regenerative small, basophilic muscle fibers with one or more centrally located nuclei (white arrows) were observed in all hADSC groups. Scale bar: 100 μm (× 20 magnification)

We started the evaluation of the limb motor functions on the second day after the artery ligation (Fig. 2a). Depending on the degree of use of the damaged limb by the mouse during movement, the score was 3, 2, 1, and 0 points [11]. Based on that assessment, it was found that mice receiving hADSCs, starting on the third day after the procedure, began to support the gait on the injured limb, whereas in the control mice the limb was constricted and impaired. On day 14, we observed only slight differences in the movement of the mice of both groups and the motor functions of the damaged limbs were comparable to the healthy ones.

### The MR angiography of the vascular system in the damaged limb after the hADSCs implantation

In order to investigate changes in the vessels in the ischemic limb, we utilized an angiographic examination. The study was carried out on days 0, 3, 7, and 14. The test on day 0 (day of arterial ligation) confirmed the efficiency of ligation of the femoral artery (Fig. 2b, f). During the study, we observed no significant differences in the angiograms of the injured limb in both groups of mice. On day 14, the artery system after ligation was similar to the artery system in the healthy limb, in the group of mice that received hADSCs as well as in PBS^−^ (Fig. 2e, i).

### The administration of hADSCs accelerates the repair of the damaged muscle

To show the changes in muscle tissue after the hADSCs administration, we collected the tissue for histochemical staining procedure on days 3, 7, 14, and 21. In the control muscles, necrotic muscle fibers were observed up to day 14 (Fig. 2j, k, l). One week after the administration of hADSCs, we observed extensive muscle regeneration (regenerative small, basophilic muscle fibers with one or more centrally located nuclei) (Fig. 2o). On day 21, the muscles treated with hADSCs and the control muscles resembled healthy tissues—the fibers were typically polygonal and the sarcolemmal nuclei were located peripherally (Fig. 2m, r).

### The administration of hADSCs increases macrophages infiltration in the mouse model of hindlimb ischemia as compared to mMSC

**Fig. 3:**
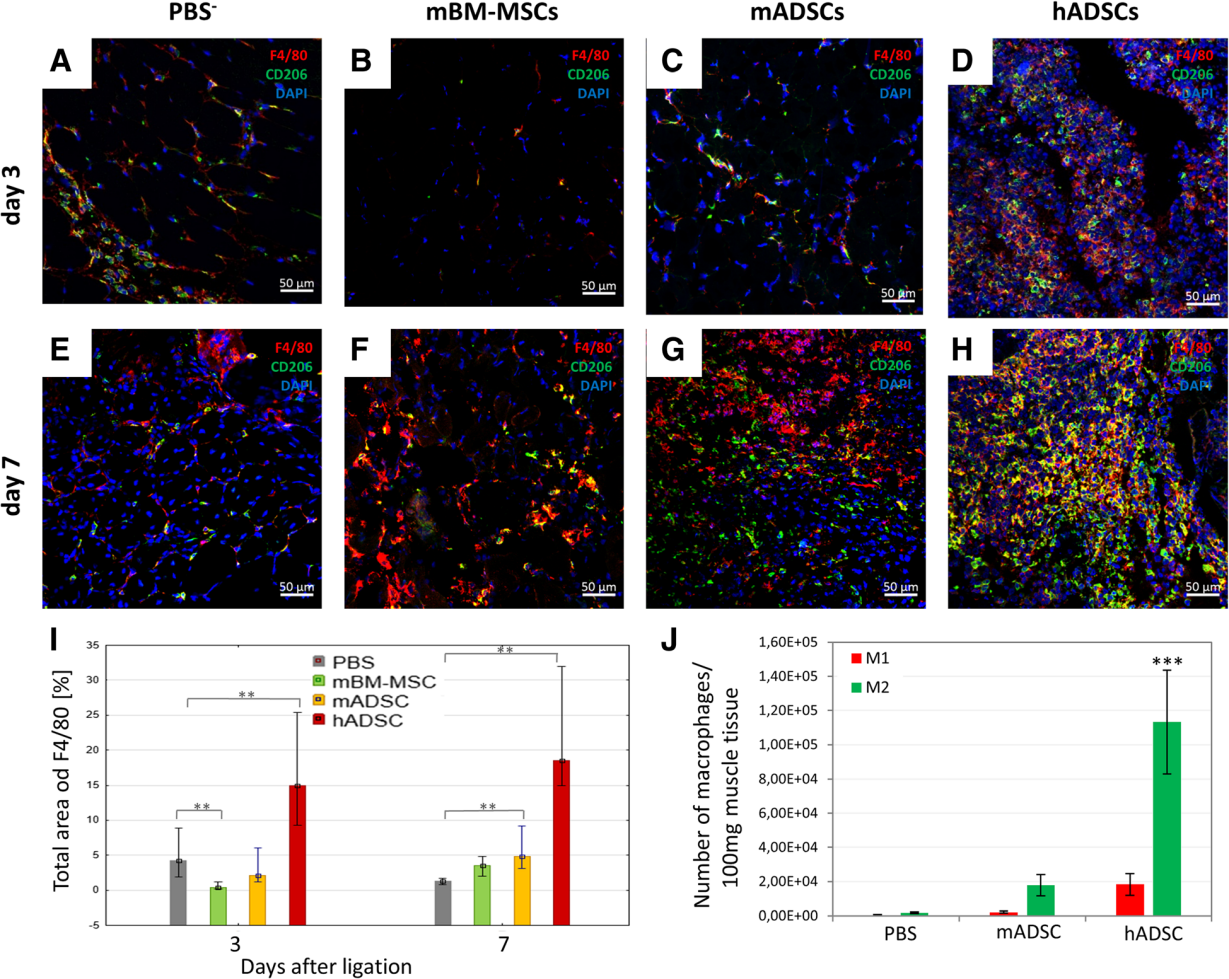
hADSCs injection in the ischemic muscles increased the infiltration of the proangiogenic M2 macrophages. Immunofluorescence analyzes in collected muscles 3 (**a**, **b**, **c**, **d**) and 7 (**e**, **f**, **g**, **h**) days after the ligation of the artery and the administration of hADSC (**d**, **h**), mADSC (**c**, **g**), mBM-MSC (**b**, **f**), and control (PBS^−^) (**a**, **e**). The representative images of M2 macrophages (F4/80^+^ (red) / CD206^+^ (green)). The images show that after the hADSC injection into ischemic muscle the infiltration of the proangiogenic macrophages M2 was observed on the 3rd day after the surgery and it increased at day 7. After the administration of mBM-MSC and PBS^−^, the significant M2 macrophage infiltration was not observed. However, after the administration of mADSC an increase in macrophage infiltration was observed on day 7. The graph of the total area of F4/80 macrophages in collected muscles on the 3rd and 7th day after the surgery (I). The total area occupied by F4/80 macrophages was calculated using ImageJ software in 10 fields of view at a magnification of 40 × (*n* = 5; 5 muscles per group were analyzed; 10 pictures of each muscle were taken). Using the flow cytometry 7 days after the artery ligation and administration of the hADSCs, mADSCs, and PBS^−^ in collected muscles the number of M1 (defined as 7-AAD^−^/CD45^+^/F4/80^+^/ CD86^+^) and M2 (defined as 7-AAD^−^/CD45^+^/F4/80^+^/CD206^+^) macrophages was evaluated (**j**). In all researched groups we observed a greater number of M2 macrophages in comparison to M1 macrophages. The largest number of M2 macrophages was found in muscles after the administration of hADSCs: six times more than mADSCs group and sixty times more than PBS^−^ group. The ratio of M2 to M1 macrophages was 686 in hADSCs group, 824 in mADSCs group and 364 in PBS^−^ group (*n* = 5). **p < 0.001 evaluated with Kruskal-Wallis one-way analysis of variance and multiple comparison of mean ranks for all groups. ***p < 0.0005 by the ANOVA followed by the Tukey’s post hoc test. Nuclei stained with DAPI (blue), scale bars = 50 μm

In the muscles, 3 days after the administration of hADSCs, we observed a significant increase in the area occupied by cells expressing F4/80 (immunofluorescence; 15.23% of the area) as compared to the control (PBS^−^) (4.29% of the area) and both murine MSC groups: mADSCs (1.97% of the area) and mMB-MSCs (0.52% of the area). On day 7, the areas of F4/80 positive cells in hADSCs group were increased to 18.62% of the total area, mADSCs group—4,88%, and mBM-MSCs group—3.23%, while in PBS group was 1.19% of the total area. Almost all macrophages (F4/80^+^ cells) also possessed the CD206 antigen (M2 macrophages) (Fig. 3). Additionally, we conducted a cytometric analysis of the macrophages in the muscles on the seventh day after the artery ligation. In all three groups (hADSCs, mADSCs and PBS^−^), we identified a larger number of M2 macrophages (defined as 7-AAD^−^/CD45^+^/F4/80^+^/CD206^+^) than M1 macrophages (defined as 7-AAD^−^/CD45^+^/F4/80^+^/CD86^+^). In the muscles of the mice which were injected with both hADSC and mADSC, we observed an increased number of M2 macrophages in comparison to the control group (hADSC—over 60-fold higher; mADSC—almost 10-fold higher) (Fig. 3j).

### The administration of hADSCs increases angiogenesis in the murine model of hindlimb ischemia

**Fig. 4:**
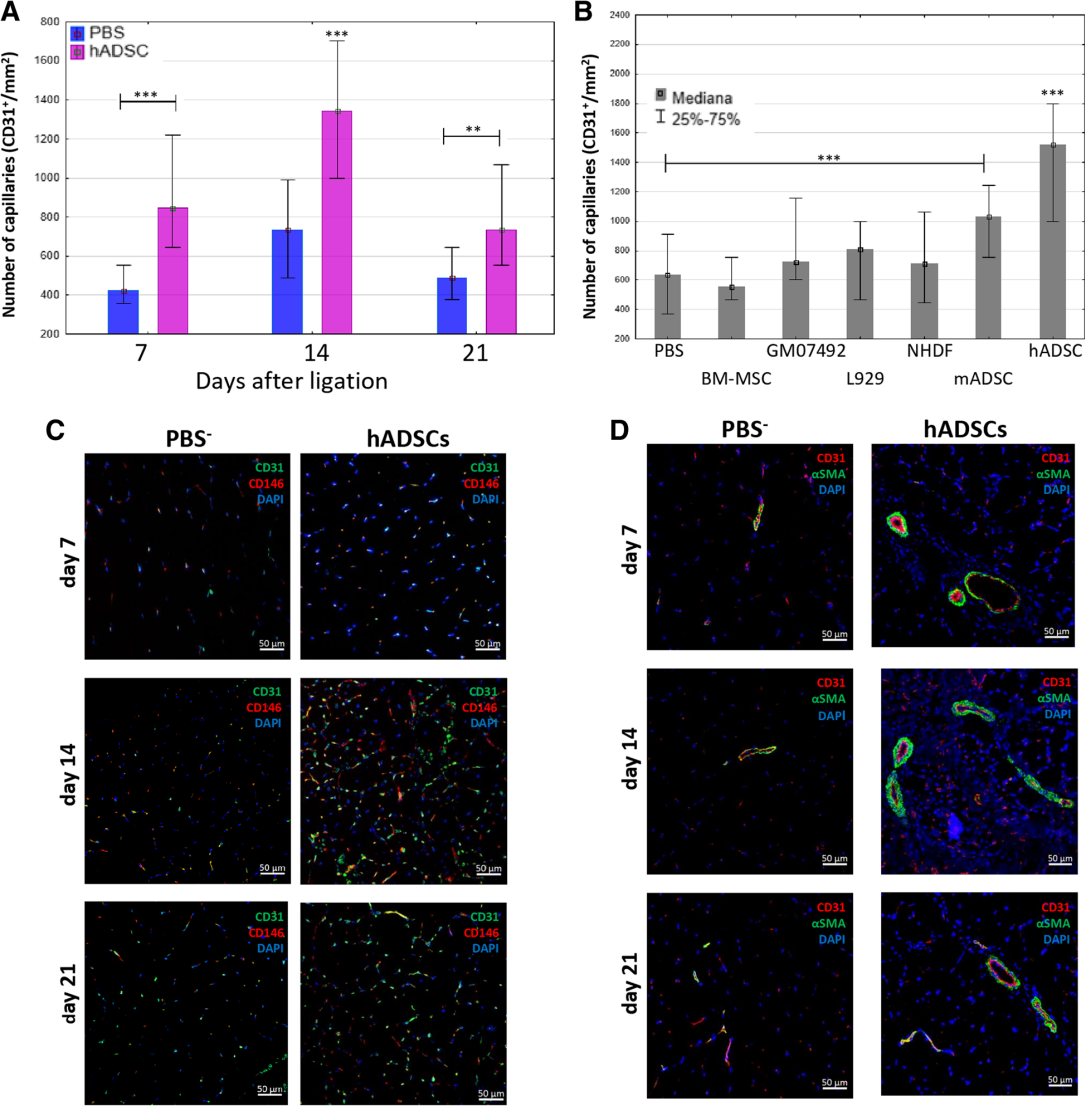
The administration of hADSCs increases the angiogenesis in mouse model of the hindlimb ischemia. Capillaries (CD31 positives cells) count in collected muscles 7, 14, and 21 days after the artery ligation and the administration of hADSC or PBS^−^ (**a**). The Number of capillaries in collected muscles 14 days after the artery ligation and the administration of hADSC, mADSC, mBM-MSC, L929, GM07492, NHDF, and PBS^−^ (**b**). The number of the vessels (CD31^+^) in each group was determined in 10 fields of view at a magnification of 40x and calculated per 1mm^2^ of tissue (*n* = 5; 5 muscles per group were analyzed; 5 (for total area of F4/80) or 10 (for number of capillaries) pictures of each muscle were taken). **p < 0.05, ***p < 0.001 evaluated with Kruskal-Wallis one-way analysis of variance and multiple comparison of mean ranks for all groups (hADSCs statistically significant with each group except the mADSC group, *p* = 0.235). Nuclei stained with DAPI (blue), scale bars = 50 μm. The immunostaining of pericytes CD146 (red)/endothelial cells (green) with nuclei staining (blue) (**c**). The immunostaining of αSMA (green)/endothelial cells (red) with nuclei staining (blue) (**d**)

The administration of hADSCs into ischemic muscle significantly increased the number of newly formed capillaries compared to the control group (PBS^−^) (Fig. 4a). By the seventh day, the number of new microvessels (CD31^+^) was twice as high (848/mm^2^) as after the administration of PBS^−^ (424/mm^2^). Until the 14th day after the surgery, the number of microvessels after hADSCs administration increased significantly (1343/mm^2^) compared to day 7 and was almost twice as high as in the control group (726/mm^2^). After 3 weeks, the number of microvessels in both groups decreased. However, a significant difference was still observed. In the group in which hADSCs were administered, the number of microvessels was higher (726/mm^2^) than in the control group (475/mm^2^). The newly formed microvessels present in the muscles were composed of endothelial cells and pericytes surrounding them (CD146^+^) (Fig. 4c). In turn, the amount of large blood vessels (α-SMA^+^) was the highest on day 7 after the administration of the hADSCs (1.13% of the area), compared to the control where we observed only few large blood vessels (0.19% of the area) (Fig. 4d).

We also examined the proangiogenic properties of murine MSC and fibroblasts, both murine and human (Fig. 4b). None of the three fibroblast cell lines (L929, GM07492, NHDF) increased the number of blood vessels in the gastrocnemius muscle on the 14th day after the surgery. In the group, where the murine MSC isolated from the bone marrow were injected into the hindlimb, we observed comparable number of blood vessels (556/mm^2^) to the control group (636/mm^2^). The administration of both murine and human MSCs isolated from adipose tissue significantly increased the number of blood vessels on the 14th day compared to the control group (PBS^−^). After the administration of hADSCs in the gastrocnemius muscle, 106% more vessels (1406/mm^2^) were observed, and after administration of mADSCs, 55% more blood vessels (1060/mm^2^) were observed as compared to the control group.

### Injected hADSCs secrete interleukin 6 and cause infiltration of macrophages in murine ischemic gastrocnemius muscle

**Fig. 5:**
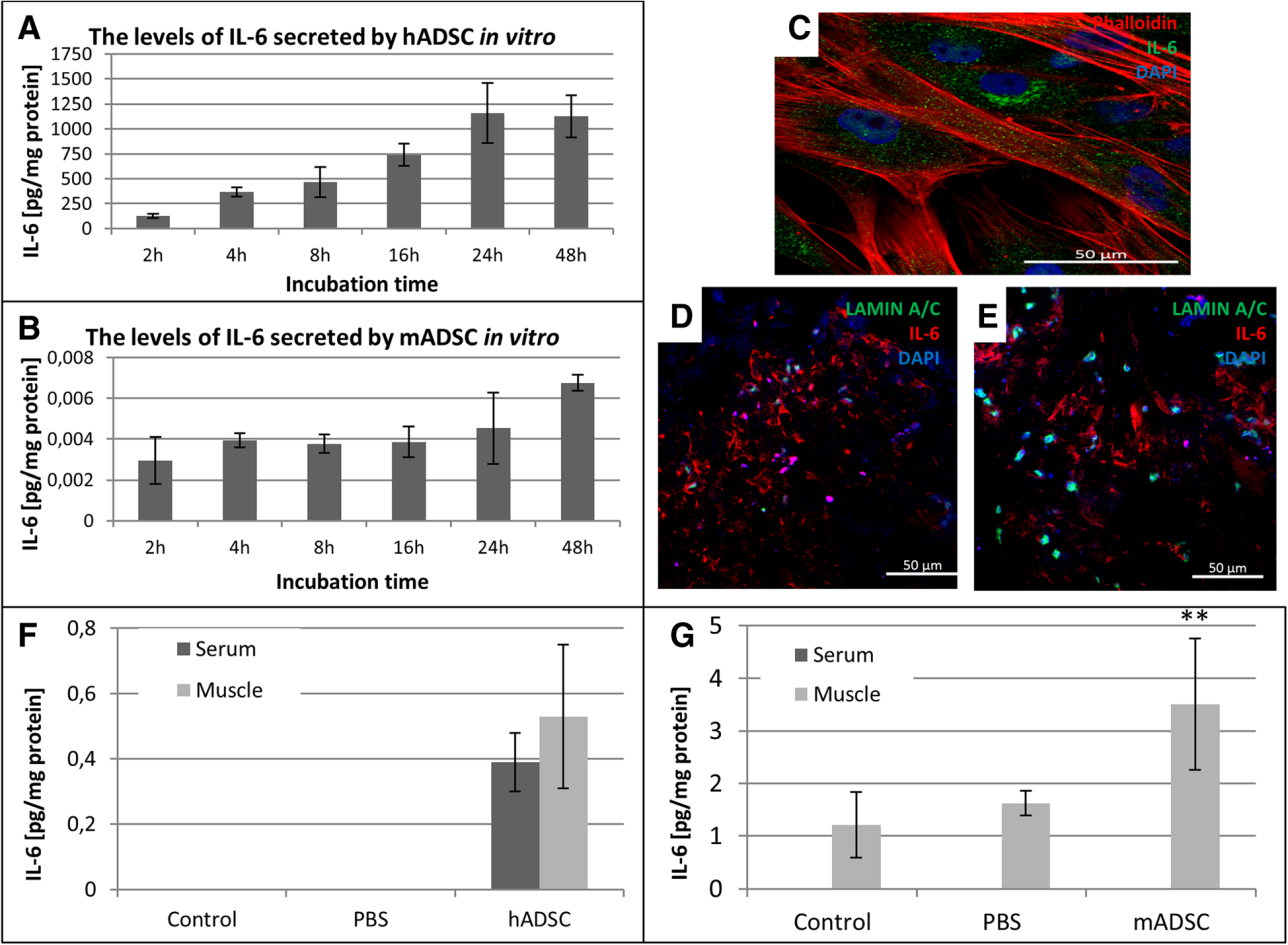
hADSCs secrete interleukin 6. The levels of IL-6 secreted by hADSCs (**a**) and mADSCs (**b**) in vitro were determined by ELISA. The amounts of secreted IL-6 were calculated for 1 mg of total protein content. The immunofluorescence staining of interleukin 6 (green) and hADSCs (red) (**c**). The representative images of the double immunofluorescence staining of human specific Lamin A/C (green) and interleukin 6 (red) (**d**, **e**). The images show that the injected hADSC localized in clusters, presumably at the injection site, and interleukin 6 was located around hADSCs on the third (**d**) and the seventh (**e**) day after the cell injection in the ischemic limb muscles. The amounts of the secreted IL-6 by human ADSCs (**f**) and murine ADSCs (**g**) in the murine muscle and serum was determined by ELISA test on the 3rd after artery ligation (*n* = 5). **p < 0.01 evaluated with *U* Mann-Whitney test. Nuclei were stained with DAPI (blue), scale bars = 50 μm

Before administering hADSCs to mice, we checked whether these cells secrete interleukin 6. The levels of IL-6 secreted in vitro by both hADSCs and mADSCs increased in a time-dependent manner (Fig. 5a, b). The amount of secreted IL-6 by murine ADSCs was significantly lower compared to human ADSCs. Immunohistochemical staining showed that interleukin 6 was located in hADSCs in vitro (Fig. 5c). Interleukin 6 was located around hADSCs after injection into the muscle (Fig. 5d, e). We investigated the amount of the secreted IL-6 in vivo after the ADSCs administration. The amount of IL-6 secreted in the gastrocnemius muscle after administering the murine ADSCs was sevenfold higher compared to hADSC (Fig. 5f, g). We observed slightly more of IL-6 in the muscles than in the serum after the administration of hADSCs. However, after administering the murine ADSCs, we did not found IL-6 in the serum (Fig. 5g). Furthermore, at the site of administration of hADSCs, we observed the M2 macrophages (CD206^+^) infiltration (Fig. 1e–g).

### The depletion of macrophages suppressed the therapeutic effect induced by hADSCs

**Fig. 6:**
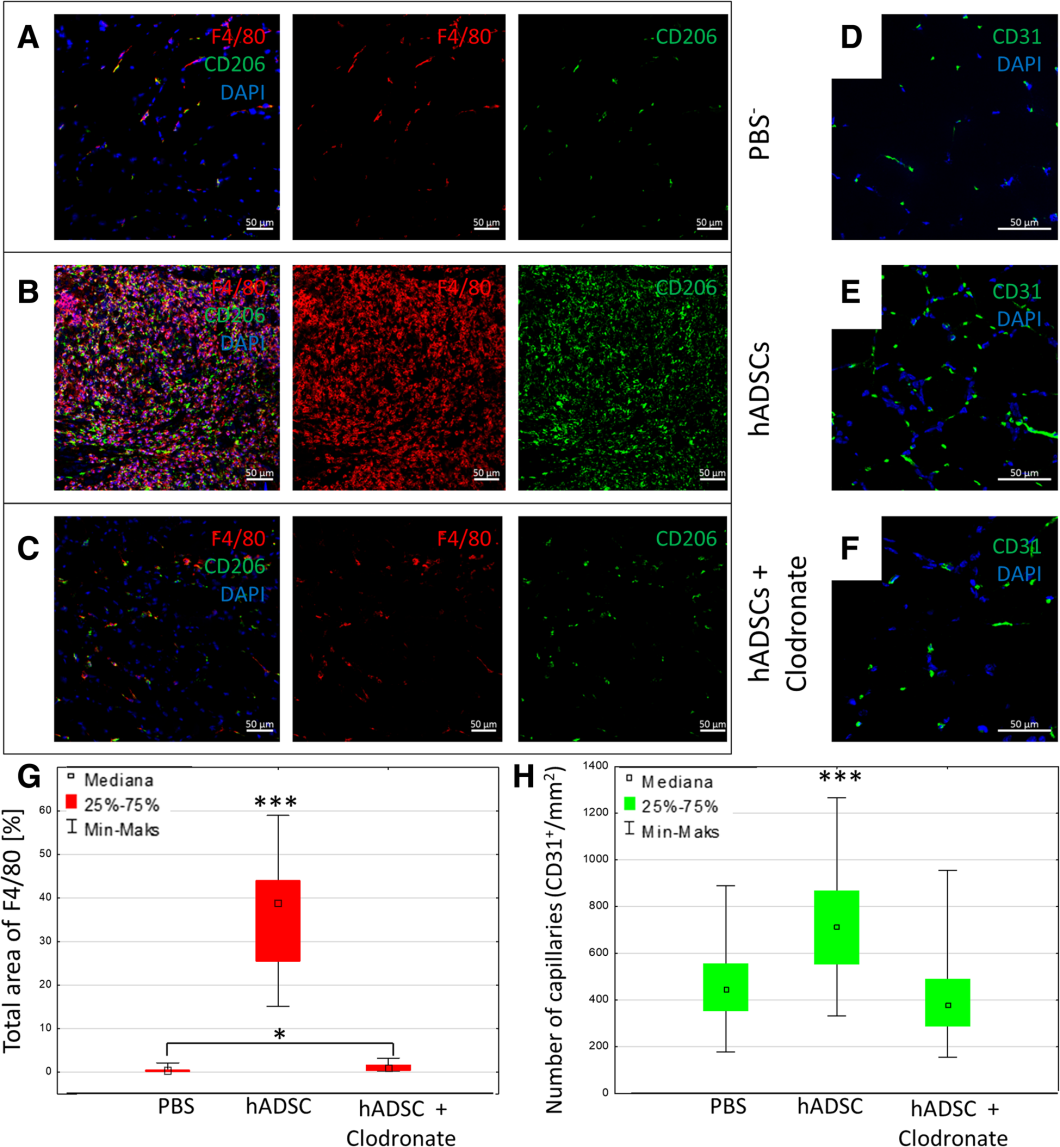
Depletion of macrophages using liposomes with clodronate abolishes the therapeutic effect induced by hADSCs. The immunofluorescence analyzes of blood vessels and macrophages 7 days after the artery ligation and hADSCs and clodronate liposomes administration (hADSCs+clodronate liposomes) and control group (hADSCs+PBS liposomes). The representative images of M2 macrophages (F4/80^+^ (red)/CD206^+^ (green)) (**a**, **b**, **c**) and blood vessels CD31^+^ (green) (**d**, **e**, **f**). The graph of the total area of F4/80 macrophages (**g**) and capillaries (CD31) (**h**) in collected muscles. The images and the graph show that liposomes containing clodronate effectively deplete F4/80^+^ and F4/80^+^CD206^+^ cells in mouse gastrocnemius muscles in vivo (**c**), (**g**). After the depletion of macrophages an increase in the number of blood vessels was not observed in muscles where hADSC were administrated (**f**), (**h**) (*n* = 10; 10 muscles per group were analyzed; 5 (for the total area of F4/80) or 10 (for the number of capillaries) pictures of each muscle were taken). *p < 0.05, ***p < 0.001 evaluated with Kruskal-Wallis one-way analysis of variance and multiple comparison of mean ranks for all groups. Nuclei stained with DAPI (blue), scale bars = 50 μm

We investigated the role of macrophages in the regeneration processes in damaged muscles after the hADSCs administration. For this purpose, we used liposomes with hADSCs with clodronate to deplete macrophages in vivo. While the hADSCs injection increased the number of F4/80^+^/CD206^+^ cells (macrophages M2) in the gastrocnemius muscle (Fig. 6b), injection of clodronate liposome reduced the infiltration of macrophages (Fig. 6c). The number of F4/80^+^ cells was significantly higher (38.69% of the area) in hADSCs group as compared to the hADSCs with clodronate liposome group (0.75% of the area) and PBS^−^ with clodronate liposome group (0.03% of the area) (Fig. 6g). Additionally, we observed no increase in the number of newly formed blood vessels (CD31^+^) in hADSCs with clodronate liposomes group (379/mm^2^) compared to hADSCs group (721/mm^2^) (Fig. 6h).

### The administration of hADSCs in a model of hindlimb ischemia in NOD SCID mice increases angiogenesis but does not regenerate the muscle

**Fig. 7:**
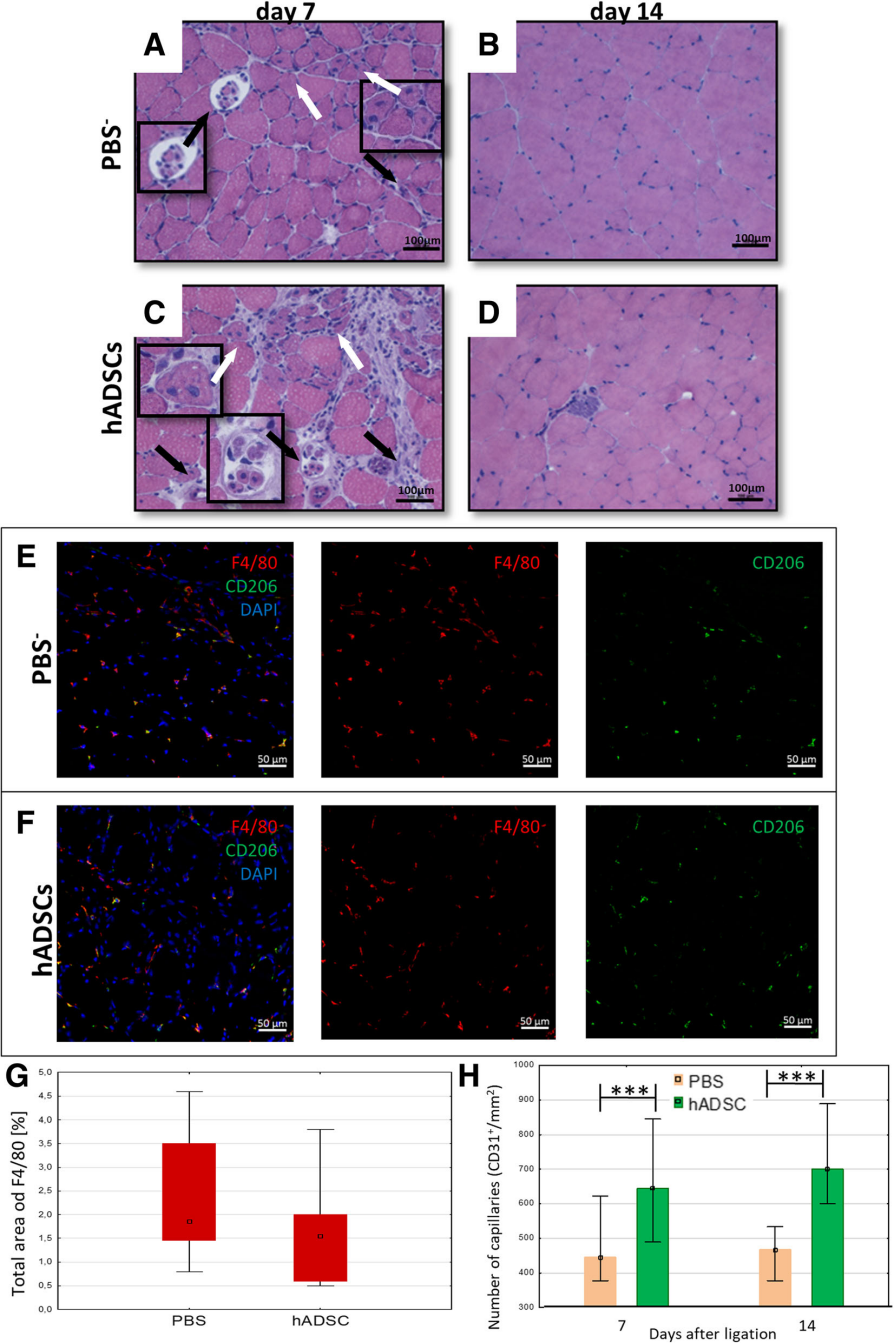
The administration of the hADSCs into the ischemic muscle of immunodeficient NOD SCID mice increases the number of new blood vessels but does not increase the macrophages infiltration. The representative images of H&E staining of the transverse sections of gastrocnemius muscle after PBS^−^ (**a**, **b)** and hADSCs (**c**, **d**) injection on days 7 and 14 post surgery. Necrotic muscle fibers (black arrows) with pale cytoplasm and regenerative small, basophilic muscle fibers with one or more centrally located nuclei (white arrows) were observed at 7 days after PBS^−^ and hADSCs administration (**a**, **c**). The representative images of M2 macrophages (F4/80^+^ (red)/CD206^+^ (green)) after the PBS^−^ (**e**) and hADSCs (**f**) injection 7 days after surgery. Seven days after the hADSCs injection into ischemic muscle of immunodeficient NOD SCID mice, the infiltration of macrophages was not observed. The graph of the total area of F4/80 macrophages shows that there is no significant difference in the macrophages presence in the muscles between PBS^−^ and hADSCs groups (**g**). The number of capillaries (CD31^+^) in collected muscles 7 and 14 days after hADSCs and PBS^−^ administration (**h**). *n* = 5; 5 muscles per group were analyzed; 5 (for the total area of F4/80) or 10 (for the number of capillaries) pictures of each muscle were taken. *p < 0.05, ***p < 0.001 evaluated with *U* Mann-Whitney test. Nuclei stained with DAPI (blue), scale bars = 50 μm

After the administration of hADSCs into ischemic muscle of NOD SCID mice, we did not observe the muscle regeneration on days 7 (Fig. 7a, c) and 14 (Fig. 7b, d). We observed the necrotic muscle fibers in the control muscles (Fig. 7a, c). Moreover, macrophages infiltration in both hADSCs and PBS^−^ groups was not observed (Fig. 7e, f, g). However, we observed an increase in the number of newly formed blood vessels (CD31^+^) after the hADSCs administration on days 7 and 14 (compared to the control group) (Fig. 7h).

### Blocking IL-6 secretion by hADSCs in vivo suppresses the therapeutic effect induced by these cells

**Fig. 8:**
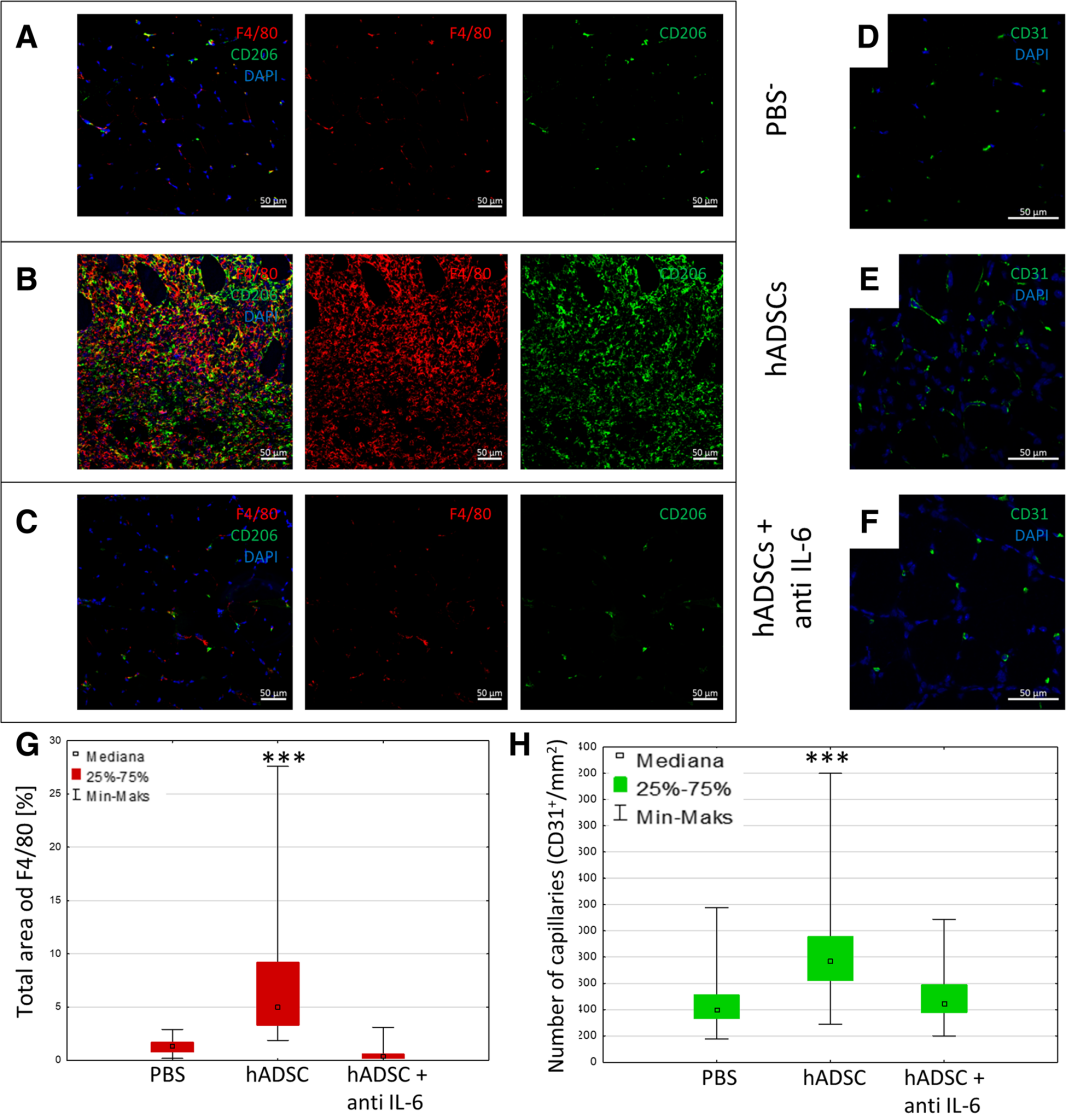
IL-6 produced by hADSCs causes an infiltration of the proangiogenic and immunosuppressive M2 macrophages and increases the number of blood vessels. The immunofluorescence analysis of the muscles collected 7 days after the artery ligation and the administration of hADSCs with human IL-6 blocking antibody (hADSCs + anti IL-6) and the control group (PBS^−^). The representative images of M2 macrophages (F4/80^+^ (red)/CD206^+^ (green)) (**a**, **b**, **c**) and blood vessels CD31^+^ (green) (**d**, **e**, **f**). After the administration of IL-6 producing hADSCs the increased infiltration of M2 macrophages (**b**) and the increase of blood vessels (**e**) were observed. After blocking of the IL-6 secreted by hADSCs in vivo no macrophage infiltration or increase in the number of blood vessels was observed (**c**, **f**). The number of capillaries (CD31^+^) (**h**) and the total area of F4/80 macrophages (**g**) counted in collected muscles 7 days after the administration of hADSC, hADSCs with siltuximab and PBS. *n* = 5; 5 muscles per group were analyzed; 5 (for the total area of F4/80) or 10 (for the number of capillaries) pictures of each muscle were taken. ***p < 0.001 evaluated with Kruskal-Wallis one-way analysis of variance and multiple comparison of mean ranks for all groups. Nuclei stained with DAPI (blue), scale bars = 50 μm

Human interleukin 6 secreted in vivo was blocked using siltuximab. After in vivo blocking of the IL-6, we did not observe the macrophages infiltration 7 days after the administration of the hADSCs (Fig. 8c). We observed a significant decrease in the area occupied by cells expressing F4/80 in gastrocnemius muscles after the injection of hADSCs and after the administration of siltuximab (0.39% of the area) as compared to the group receiving the cells alone (32.3% of the area) (Fig. 8g). Likewise, in groups with siltuximab and hADSCs, we did not observe an increase in the number of newly formed blood vessels (CD31^+^) (432/mm^2^) compared to group receiving hADSCs alone (736/mm^2^) (Fig. 8h).

### The administration of interleukin 6 alone has no therapeutic effect in mouse model of hindlimb ischemia

**Fig. 9:**
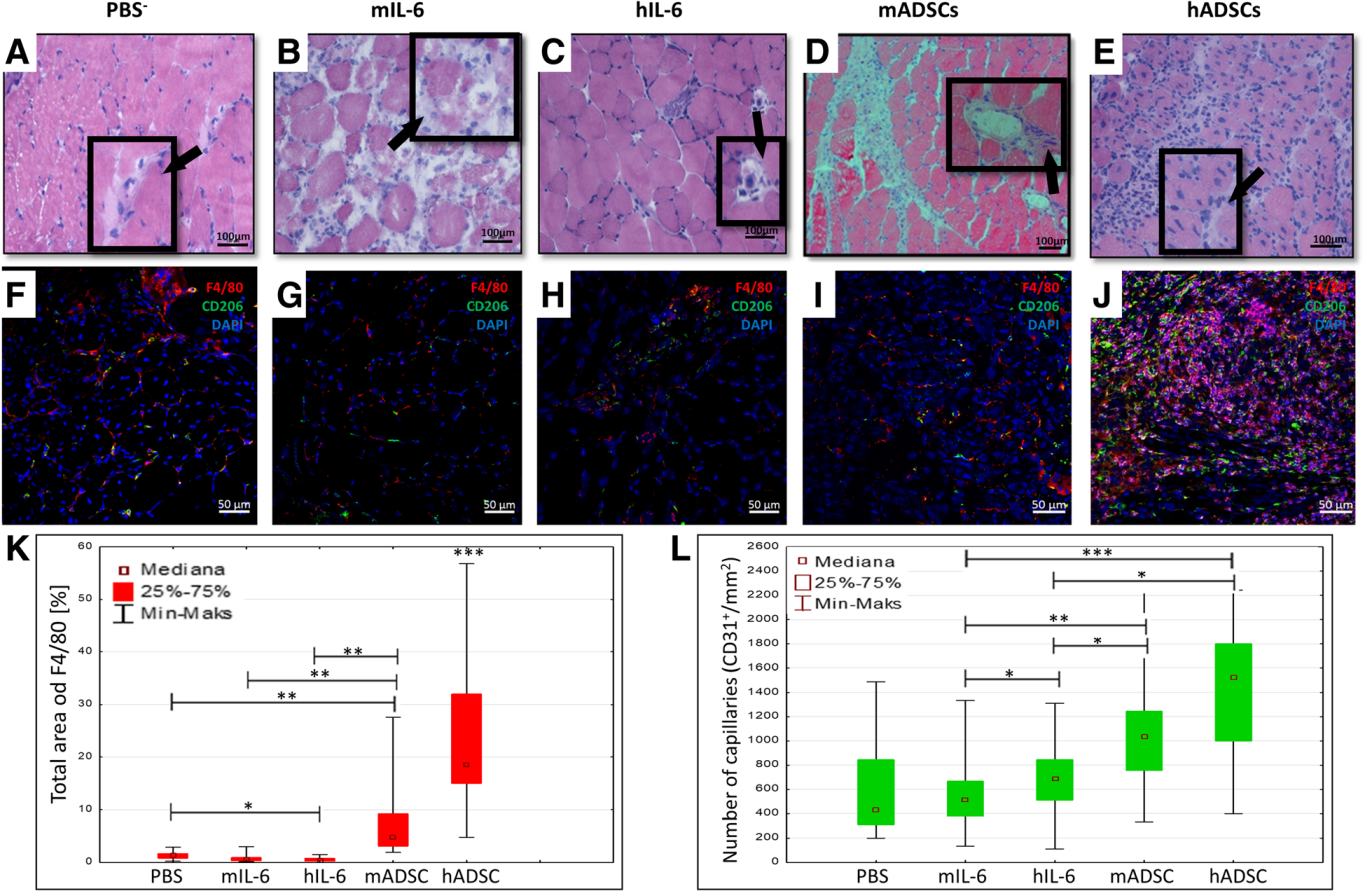
The administration of interleukin 6 into ischemic muscle increases the number of new blood vessels but not the macrophages infiltration. The representative images of H&E staining of the transverse sections of the gastrocnemius muscle after PBS^−^ (**a**), mouse recombinant interleukin 6 (**b**), human recombinant interleukin 6 (**c**), mADSC (**d**), and hADSC (**e**) injection on day 7 post surgery. Several necrotic muscle fibers (black arrows) were observed in muscles after the administration of PBS^−^ (**a**) human recombinant interleukin 6 (**c**) and mADSC were observed? (**d**). Many necrotic muscle fibers with pale cytoplasm and the irregular internal architecture were observed in muscles after the administration of mouse recombinant interleukin 6 (**b**). Regenerative small muscle fibers with one or more centrally located nuclei (white arrows) were observed only in the muscles after hADSCs administration (**d**). The representative images of M2 macrophages (F4/80^+^ (red)/CD206^+^ (green)) after PBS^−^, mIL-6, hIL-6, mADSC, and hADSC injection on the seventh day after the surgery (**f**, **g**, **h**, **i**, **j**). The number of capillaries (CD31^+^) (**l**) and the total area of F4/80 macrophages (**k**) counted in collected muscles 7 days after the administration of hADSC, mouse and human recombinant interleukin 6, and PBS. *n* = 5; 5 muscles per group were analyzed; 5 (for the total area of F4/80) or 10 (for the number of capillaries) pictures of each muscle were taken. *p < 0.05, ***p < 0.001 evaluated with Kruskal-Wallis one-way analysis of variance and multiple comparison of mean ranks for all groups. Nuclei stained with DAPI (blue), scale bars = 50 μm

We checked whether the administration of interleukin 6 alone would induce a similar therapeutic effect as the administration of IL-6 secreting hADSCs and mADSCs cells. For this purpose, we administered recombinant human or murine IL-6 proteins to mice. We observed muscle regeneration (regenerative small muscle fibers with one or more centrally located nuclei) 7 days after the hADSCs administration (Fig. 9e). No muscle regeneration was observed after PBS^−^ (Fig. 9a), murine interleukin 6 (mIL-6) (Fig. 9b), human interleukin 6 (hIL-6) (Fig. 9c), and mADSCs (Fig. 9d) administration. Additionally, after the mIL-6 injection, we observed extensive muscle degeneration (many necrotic muscle fibers with irregular internal architecture). The administration of murine interleukin 6 into muscle did not increase the number of new blood vessels (543/mm^2^), but the administration of human interleukin 6 (hIL-6) increased significantly the number of new blood vessels by 29% (682/mm^2^) as compared to the control group (PBS) on the seventh day after the surgery. However, the number of new blood vessels after the hADSCs administration was 24% higher compared to human IL6 and after mADSCs administration, it was twice as high compared to murine IL-6 (Fig. 9l). After the administration of mIL-6 and hIL-6, twice as many macrophages, respectively, 0.695% of the area and 0,615% of the area as compared to control group (PBS^−^) (0.32% of the area) were observed. However, this amount of macrophages was significantly lower compared to hADSC group (32.3% of the area) and mADSCs group (4.88% of the area) (Fig. 9k).

## Discussion

The aim of our work was to explain the mechanism of the repair of the damaged muscle in the hind limb ischemia model with the participation of human adipose-derived mesenchymal stromal cells (hADSCs).

The isolation of mesenchymal cells from adipose tissue has many advantages. Adipose tissue collection, most often during liposuction, is safe and easy, and the isolation of cells from collected samples is highly efficient [5]. It is estimated that it is about five times more efficient than, for example, isolation from the bone marrow [12]. Due to the properties of ADSCs, there have been many researches looking for the possibility of using it in different types of therapies connected to the repair of the damaged tissues [13–16].

hADSCs, depending on the environmental context, show anti- or proinflammatory properties [17–21]. They interact with macrophages [21], T and B lymphocytes [22], and NK cells [23]. Therefore, in our studies, we decided to use two mice strains: immunocompetent C57BL/6NCrl strain and NOD SCID strain with defective immune system.

In the first stage of the study, we examined the retention time of hADSCs transferred into the muscle of C57BL/6NCrl mice. Fourteen days after administration, the cells were still present in the muscle (data not shown). In the study by Dao et al. [24], the retention time of hADSCs in murine ischemic limb was shorter and lasted 8 days. The differences in the assessment of the retention time in both studies may be due to different methods of cells identification in the tissue.

After the procedures of artery ligation and administration of hADSCs, the condition of the mice, especially the mobility of the damaged limb, were evaluated daily. We observed that on the third day after the surgery the gait of mice that received hADSCs was supported by the damaged limb and in control mice the limb was still impaired. On day 14 after surgery we only observed slight differences in the movement ability of mice of both groups (Fig. 2a).

A similar assessment was presented in the article [11] in which therapy with the use of mouse mesenchymal cells isolated from the bone marrow was conducted. On day 14, we observed the normal morphology of muscle fibers (Fig. 2m, r). It confirms that the process of the repair of the damaged limb after artery ligation takes about 2 weeks and administration of hADSCs accelerates this process.

We observed a higher number of newly formed vessels in the injured muscle to which the hADSCs were administered than in the control mice. The number of newly formed capillaries was changing during the study. The highest number of blood vessels was noted on day 14 after the hADSCs administration and then the number decreased on day 21 to a level slightly lower than on day 7. We observed a similar process in the group of control mice that received PBS. The number of capillaries was the highest on day 14 to then decrease on day 21 to the level from day 7. On day 21, the number of capillaries in the hADSCs group was higher than that in the PBS^−^ group (Fig. 4a). Similarly, to others [25], we observed that this reduction in the number of capillaries is the result of the formation of large, functional blood vessels and the stabilization of the microenvironment in the limb.

We observed infiltration of macrophages with the M2 phenotype characterized by the proangiogenic and immunosuppressive properties in the vicinity of the administered hADSCs.

We should remember that the population of the M2 macrophages is not homogeneous and may be divided into three main subtypes: M2a, M2b, and M2c [26]. In our work, we evaluated the number of M1 and M2 macrophages without the exact characteristic of the M2 macrophages population according to their subtypes: mainly M2a and M2b.

Similar infiltration of macrophages is described in the publication [27]. In that work, however, the phenotype of the incoming macrophages had not been determined. M1 macrophages are proinflammatory and cytotoxic while M2 macrophages are immunosuppressive and proangiogenic. In our opinion, the authors of the study, like us, concluded that the infiltration of M2 macrophages is responsible, among others, for the healing process.

Summarizing, in the muscle after treatment with hADSCs, a larger infiltration of macrophages with the M2 phenotype and more capillaries are identified in the muscles than in the muscles of the control mice.

In our work [28], we observed that IL-6 is a dominant cytokine secreted by mesenchymal cells isolated from the myocardium. IL-6 plays a significant role in the repair of the damaged tissues. We decided to determine its level in the hADSCs culture medium (Fig. 5a). A similar analysis was also carried out for murine MSCs isolated from adipose tissue (Fig. 5b) and bone marrow. hADSCs secreted more IL-6 compared to other cells. In vivo, the presence of IL-6 was confirmed in the immediate vicinity of hADSCs (Fig. 5d, e), which may prove that this cytokine is secreted by the mesenchymal cells into the environment of the damaged muscle.

IL-6 is involved in the polarization of M1 proinflammatory macrophages to immunosuppressive and proangiogenic M2 macrophages [7, 8]. The human IL-6 is active in the murine cells. Human and murine interleukin 6 show 65% of homology on the nucleotide level and 41% of homology on the amino acid level. A high level of homology between mIL-6 and hIL-6 in the region of the signal peptides was also observed; however, the end sequences of the polypeptide chain containing the amino group are different in both species [29]. We found the infiltration of M2 macrophages with IL-6 receptor expression in the vicinity of hADSCs (data not shown).

According to our hypothesis, the M2 macrophages are activated in the muscle under the influence of IL-6 secreted by hADSCs. A similar hypothesis is proposed by the authors in the work [30] in which they prove that human mesenchymal cells transform macrophages into M2 phenotype. The M2 macrophages are involved in the inhibition of the inflammatory response and initiate repair processes of damaged muscle tissue. To confirm this assumption, we had planned a few experiments involving the exclusion of one of the elements of the proposed mechanism. In the first experiment, we performed the depletion of macrophages with the use of liposomes with clodronate. In the group of mice that were administered with hADSCs together with clodronate liposomes, the number of newly formed vessels was lower than in mice administered with hADSCs with control liposomes and higher than the number of vessels in mice treated only with PBS (Fig. 6h).

We observed a similar effect in NOD SCID mice. This mouse strain does not have T and B lymphocytes and macrophages, NK cells. The dendritic cells are impaired. Similarly, to the clodronate study, we also observed a greater number of blood vessels than in the PBS control group (Fig. 7h) but definitely lower than that in C57BL/6NCrl mice. What is important, we did not observe the repair of the damaged muscle fibers (Fig. 7b, d). A greater number of vessels in the group of NOD SCID mice than in the control group is probably connected to the secretion of proangiogenic cytokines, such as VEGF, bFGF, and HGF, by the hADSC cells [30].

We need to remember that IL-6 is a cytokine with a pleiotropic effect. The literature suggest a possibility of the HGF and VEGF growth factors secretion as a consequence of JAK-STAT3 signal path activation by IL-6 [31].

In the next experiment, we used anti-human IL-6 antibody (siltuximab). The inhibition of IL-6 should suppress the M2 macrophages. We did not observe an influx of the M2 macrophages in the mice that received anti-IL-6 antibody (Fig. 8c, g). The number of newly formed blood vessels in this group of mice was lower than in the group of mice that received hADSCs alone, but comparable to the number of vessels in mice that received PBS^−^ (Fig. 8h).

Based on the experience with anti-IL-6, we asked ourselves a question: Is it sufficient to administer only the IL-6 protein in order to repair the damaged muscle or is the presence of the hADSCs necessary? We had planned an experiment in which we compared the effectiveness of murine and human IL-6 with the ADSCs administration. We compared both factors in the same experimental model. And so, the hADSCs, mADSCs, mIL-6, and hIL-6 were administered in a single dose directly into the muscle of the mouse limb an hour after the femoral artery ligation procedure.

In the mice treated with human or murine IL-6 proteins, the number of macrophages was similar to that of the mice which were administered with PBS^−^ (Fig. 9k). However, the number of newly formed vessels in the group where human IL-6 was administered was higher than that in the group where murine IL-6 was used (Fig. 9l). The highest number of incoming macrophages and newly formed vessels was observed in the group of mice treated with hADSCs.

There has not been any research so far where IL-6 was administered directly to the muscle after the femoral artery ligation, and the effect of this cytokine on the angiogenesis or the regeneration of the damaged muscle was appraised. Evaluating the gathered results, we can say that in this experimental model, with the IL-6 in 150 ng concentration, we did not observe an increased number of blood vessels. However, it is possible that the dose was too small.

According to the literature, the IL-6 is very quickly eliminated from the blood serum [32], which might have been the reason IL-6 was completely removed from the serum after a short time. Probably due to the lack of IL-6, we did not observe the activation of the M2 macrophages and the number of the blood vessels was not increased. However, the hADSCs may be treated as a some sort of “reactor” constantly producing and secreting IL-6 into the blood serum. We can presume that as long as the hADSCs are present in the muscle of the mouse, the IL-6 is present in the organism. Even though its concentration decreases with the depletion of the hADSCs, it is high enough for the several initial days to activate the macrophages.

It seems, therefore, that the longer presence of IL-6 in the blood serum is necessary for the activation of M2 macrophages. Except for the hADSCs, this effect may be achieved in two ways. The first method is to administer IL-6 multiple times in short time spans. However, taking into consideration the rate of the IL-6 degradation from the serum, it is unlikely. The second method entails an administration of the IL-6 coding plasmid DNA into the muscle of the mouse using, e.g., viruses or electroporation. The muscle cells then become, similarly to hADSC, some sort of “reactors” producing IL-6 and continuously secrete it into the blood serum. This solution seems to be more promising, as it is similar to the hADSC cell therapy. However, we are unable to say if it is going to be successful.

We need to remember that hADSCs secrete a number of agents, which activate repair processes in a damaged tissue. Those agents can be divided into six main categories: immunomodulatory (IDO, PGE2, HLA-G5, TGFβ, HGF, NO, LIF, IL-10), proangiogenic (VEGF, IGF-1, PLGF, MCP-1, bFGF, IL-6, angiopoietin-1), anti-apoptotic (VEGF, HGF, IGF-1, TGFβ, GM-CSF), countering the tissue scarring (bFGF, HGF), facilitating growth and differentiation of the resident stem cells or progenitor cells (SCF, LIF, IL-6, M-CSF, SDF-1, angiopoietin-1), and chemokines (CCL2, CCL3, CCL4, CCL5, CCL7, CCL20, CCL26, CXCL5, CXCL11, CXCL1, CXCL2, CXCL8, CXCL12) [33]. Those agents are present where hADSCs are injected, but they are absent in the microenvironment where we administer only IL-6. We do not know what effect these factors and the processes they activate could have on the mechanism we describe.

The result of the experiment with the human IL-6 does not impair its role in our proposed mechanism. It justifies the usage of the human adipose-derived mesenchymal stromal cells in the therapy. It seems that the cell to cell contact and the whole panel of the secreted cytokine, e.g., the proangiogenic cytokine (VEGF, HGF), has a significance in the repair mechanism of the damaged tissue [34].

We also found that xenografts (human ADSCs) elicited better therapeutic effects than allogenic murine MSC isolated from bone marrow and adipose tissue.

Therefore, we can pose a question: Was the therapeutic effect observed after the administration of the human MSC into the organism of a mouse a result of the action of the cells or rather was it caused by the response of the immune system to the xenograft? The results of our research and the reports in the literature [35–38] indicate that it is rather an effect of the injected mesenchymal cells, especially that the MSC are characterized by so called immunological privilege [39].

They do not possess on their surface class II MHC antigens and costimulating CD80 and CD86 antigens. They possess only small amounts of class I MHC antigens [40]. It allows them to avoid to be detected and rejected by recipient’s immune system cells [39].

However, in certain conditions (low level of IFN-*γ*), MSC may activate an immunological response [41]. Additionally, NK cells activated by IL-2 cytokine can lyse the allogenic MSC. According to Grinnmo et al., the xenografts of MSC after transplantation into the post myocardial infarction heart of a rat are rejected by the host’s organism and cause immune cells infiltration, mainly macrophages, into the site of the cells transplantation [42].

We cannot exclude a possibility that in certain conditions the immune system of the recipient is activated. It is connected rather to the status of the microenvironment where the MSC cells are introduced and not to the cells themselves.

We are convinced that the therapeutic effect which we observe in our research is caused by the administration of hADSCs. It is supported by the results of the experiment with blocking human IL-6 using sultiximab. In the group of mice with hADSCs and sultiximab administration, we did not observe an influx of M2 macrophages and increased number of blood vessels.

In the group of mice which were not administered with sultiximab, we can observe an infiltration of M2 macrophages. Therefore, we can assume that hIL-6 secreted by hADSCs is responsible for the infiltration of the M2 macrophages. If the presence of the macrophages was a consequence of the activation of the immune system of the mouse, the infiltration should be observed irrespectively of blocking hIL-6 and it would be mainly M1 macrophages. It should be noted that in our conditions the administered hADSCs live short, about 14 days, but long enough to trigger the therapeutic effect. The influence of multiple administrations of MSC cells on the immune system of the recipient still remains a separate matter.

Higher efficiency of hADSCs is probably due to the release of larger amounts of IL-6. However, we cannot exclude that it might be the cause of the activation of different subtypes of M2 macrophages by hADSC cells of allograft and xenograft. In the future, we plan to investigate the participation of M2a and M2b macrophages in the repair process of damaged tissues with the utilization of ADSC.

The goal of our therapy was not to replace the damaged muscle tissue with hADSCs which differentiated into muscle cells. The aim was to activate the mechanisms enabling the repair of the damaged tissue. Our results confirm that 14 days is a sufficient period to initiate such mechanisms in mice.

It seems that this solution may be used in clinical practice: hADSCs initiate the repair process and then die or are removed by the immune system without any risk to the recipient. Short retention time (14 days) of hADSCs in the muscle allows repeated administration of the cells which may or may not increase the therapeutic effect. Naturally, we cannot exclude the idea that multiple usage of hADSC cells can in the end cause an excessive reaction of the immune system in the organism of the recipient.

The efficiency of the cells isolation depends on the quality and origin of the collected tissue. Cells isolated from fragments of adipose tissue collected from various donors differed in their morphology and the ability to multiply in culture (unpublished results). In clinical practice, it would mean that for a particular group of patients we would not be able to obtain a sufficient number of hADSCs for cell therapy. The solution to this problem may be the hADSCs allograft. Based on our research, it seems possible and relatively safe. It also seems that stimulation of the cells to increase IL-6 secretion could improve the therapeutic effect.

## Conclusions

In conclusion, the hADSCs introduced into the damaged tissue secrete interleukin 6. In our opinion, this cytokine activates macrophages with the M2 phenotype, which suppress inflammation and activate the process of angiogenesis and the repair of the damaged muscle.

## Abbreviations

ADSCs: Adipose-derived mesenchymal stromal cells
GM07492: Human fibroblasts
hADSCs: Human adipose-derived mesenchymal stromal cells
IL-6: Interleukin 6
L929: Murine fibroblasts
mADSCs: Mice adipose-derived mesenchymal stromal cells
mBM-MSCs: Mouse bone marrow-derived mesenchymal stromal cells
MSCs: Mesenchymal stromal cells
NHDF: Human fibroblasts
PBS: Phosphate buffer saline
VEGF: Vascular endothelial growth factor

## References

[1] Liew A, O’Brien T (2012). Therapeutic potential for mesenchymal stem cell transplantation in critical limb ischemia. Stem Cell Res Ther.

[2] Henning RJ (2016). Therapeutic angiogenesis: angiogenic growth factors for ischemic heart disease. Futur Cardiol.

[3] Zhao L, Johnson T, Liu D (2017). Therapeutic angiogenesis of adipose-derived stem cells for ischemic diseases. Stem Cell Res Ther.

[4] Moon MH, Kim SY, Kim YJ, Kim SJ, Lee JB, Bae YC, Sung SM, Jung JS (2006). Human adipose tissue-derived mesenchymal stem cells improve postnatal neovascularization in a mouse model of hindlimb ischemia. Cell Physiol Biochem.

[5] Frese L, Petra E, Dijkman PE, Hoerstrup SP (2016). Adipose tissue-derived stem cells in regenerative medicine. Transfus Med Hemother.

[6] Schellera J, Chalarisb A, Schmidt-Arras D, Rose-John S (2011). The pro- and anti-inflammatory properties of the cytokine interleukin-6. Biochim Biophys Acta.

[7] Mauer J, Chaurasia B, Goldau J, Vogt MC, Ruud J, Nguyen KD, Theurich S, Hausen AC, Schmitz J, Brönneke HS, Estevez E, Allen TL, Mesaros A, Partridge L, Febbraio MA, Chawla A, Wunderlich FT, Brüning JC (2014). Signaling by IL-6 promotes alternative activation of macrophages to limit endotoxemia and obesity-associated resistance to insulin. Nat Immunol.

[8] Sanmarco LM, Ponce NE, Visconti LM, Eberhardt N, Theumer MG, Minguez ÁR, Aoki MP (2017). IL-6 promotes M2 macrophage polarization by modulating purinergic signaling and regulates the lethal release of nitric oxide during Trypanosoma cruzi infection. Biochim Biophys Acta.

[9] Wynn TA, Vannela KM (2016). Macrophages in tissue repair, regeneration, and fibrosis. Immunity..

[10] Rossini A, Frati C, Lagrasta C, Graiani G, Scopece A, Cavalli S, Musso E, Baccarin M, Di Segni M, Fagnoni F, Germani A, Quaini E, Mayr M, Xu Q, Barbuti A, DiFrancesco D, Pompilio G, Quaini F, Gaetano C, Capogrossi MC (2011). Human cardiac and bone marrow stromal cells exhibit distinctive properties related to their origin. Cardiovasc Res.

[11] Rahman MM, Subramani J, Ghosh M, Denninger JK, Takeda K, Fong GH, Carlson ME, Shapiro LH (2014). CD13 promotes mesenchymal stem cell-mediated regeneration of ischemic muscle. Front Physiol.

[12] Fraser JK, Wulur I, Alfonso Z, Hedrick MH (2006). Fat tissue: an underappreciated source of stem cells for biotechnology. Trends Biotechnol.

[13] Fitzsimmons REB, Mazurek MS, Soos A, Simmons CA. Mesenchymal stromal/stem cells in regenerative medicine and tissue engineering. Stem Cells Int. 2018:8031718. 10.1155/2018/8031718. 10.1155/2018/8031718 PMC6120267 30210552

[14] Qomi RT, Sheykhhasan M (2017). Adipose-derived stromal cell in regenerative medicine: a review. World J Stem Cells..

[15] Xu Y, Liu Z, Liu L, Zhao C, Xiong F, Zhou C, Li Y, Shan Y, Peng F, Zhang C (2008). Neurospheres from rat adipose-derived stem cells could be induced into functional Schwann cell-like cells in vitro. BMC Neurosci.

[16] Xu Y, Liu L, Li Y, Zhou C, Xiong F, Liu Z, Gu R, Hou X, Zhang C (2008). Myelin-forming ability of Schwann cell-like cells induced from rat adipose-derived stem cells in vitro. Brain Res.

[17] Bernando ME, Fibbe WE (2013). Mesenchymal stromal cells: sensors and switchers of inflammation. Cell Stem Cell.

[18] Casiraghi F, Azzollini N, Todeschini M, Cavinato RA, Cassis P, Solini S, Rota C, Morigi M, Introna M, Maranta R, Perico N, Remuzzi G, Noris M (2012). Localization of mesenchymal stromal cells dictates their immune or proinflammatory effects in kidney transplantation. Am J Transplant.

[19] Wang Y, Chen X, Cao W, Shi Y (2014). Plasticity of mesenchymal stem cells in immunomodulation: pathological and therapeutic implications. Nat Immunol.

[20] Lin T, Pajarinen J, Nabeshima A, Lu L, Nathan K, Jämsen E, Yao Z, Goodman SB (2017). Preconditioning of murine mesenchymal stem cells synergistically enhanced immunomodulation and osteogenesis. Stem Cell Res Ther.

[21] Lee HY, Hong IS (2017). Double-edged sword of mesenchymal stem cells: Cancer-promoting versus therapeutic potential. Cancer Sci.

[22] Mao F, Tu Q, Wang L (2017). Mesenchymal stem cells and their therapeutic applications in inflammatory bowel disease. Oncotarget..

[23] Spaggiari GM, Moretta L (2013). Cellular and molecular interactions of mesenchymal stem cells in innate immunity. Immunol Cell Biol.

[24] Dao TTT, Vu NB, Phi LT, Thi -Ngan Le H, Phan NK, Ta VT, Van Pham P (2016). Human adipose-derived mesenchymal stem cell could participate in angiogenesis in a mouse model of acute hindlimb ischemia. Biomed Res Ther.

[25] Landázuri N, Joseph G, Guldberg RE, Taylor WR (2012). Growth and regression of vasculature in healthy and diabetic mice after hindlimb ischemia. Am J Physiol Regul Integr Comp Physiol.

[26] Martinez FO, Gordon S (2014). The M1 and M2 paradigm of macrophage activation: time for reassessment. F1000Prime Rep.

[27] Daldrup-Link HE, Chan C, Lenkov O, Taghavigarmestani S, Nazekati T, Nejadnik H, Chapelin F, Khurana A, Tong X, Yang F, Pisani L, Longaker M, Gambhir SS (2017). Detection of stem cell transplant rejection with ferumoxytol MR imaging: correlation of MR imaging findings with those at intravital microscopy. Radiology..

[28] Czapla J, Matuszczak S, Wisniewska E, Jarosz-Biej M, Smolarczyk R, Cichoń T, Głowala-Kosińska M, Śliwka J, Garbacz M, Szczypior M, Jaźwiec T, Langrzyk A, Zembala M, Szala S. Human cardiac mesenchymal stromal cells with CD105^+^CD34^−^ phenotype enhance the function of post-infarction heart in mice. Int J Stem Cells. 2016;11(7). 10.1371/journal.pone.0158745. 10.1371/journal.pone.0158745 PMC4945149 27415778

[29] Chiu CP, Moulds C, Coffman R (1988). Multiple biological activities are expressed by a mouse interleukin6 cDNA clone isolated isolated from bone marrow stromal cells. Proc Natl Acad Sci U S A.

[30] Qiu X, Liu S, Zhang H, Zhu B, Su Y, Zheng C, Tian R, Wang M, Kuang H, Zhao X, Jin Y (2018). Mesenchymal stem cells and extracellular matrix scaffold promote muscle regeneration by synergistically regulating macrophage polarization toward the M2 phenotype. Stem Cell Res Ther.

[31] Shabbir A, Zisa D, Lin H, Mastri M, Roloff G, Suzuki G, Lee T (2010). Activation of host tissue trophic factors through JAK-STAT3 signaling: a mechanism of mesenchymal stem cell-mediated cardiac repair. Am J Physiol Heart Circ Physiol.

[32] Castell JV, Gomez-Lechon MJ, David M, Andus T, Geiger T, Trullenque R (1989). Interleukin-6 is the major regulator of acute phase protein synthesis in adult human hepatocytes. FEBS Lett.

[33] Meirelles LS, Fontes AM, Covas DT, Caplan AI (2009). Mechanisms involved in the therapeutic properties of mesenchymal stem cells. Cytokine Growth Factor Rev.

[34] Collawn SS, Patel S (2014). Adipose-derived stem cells, their secretome, and wound healing. J Cell Sci Ther.

[35] Bartholomew A, Sturgeon C, Siatskas M, Ferrer K, McIntosh K, Patil S, Hardy W, Devine S, Ucker D, Deans R, Moseley A, Hoffman R (2002). Mesenchymal stem cells suppress lymphocyte proliferation in vitro and prolong skin graft survival in vivo. Exp Hematol.

[36] Tse WT, Pendleton JD, Beyer WM, Egalka MC, Guinan EC (2003). Suppression of allogeneic T-cell proliferation by human marrow stromal cells: implications in transplantation. Transplantation..

[37] Saito T, Kuang JQ, Bittira B, Al-Khaldi A, Chiu RC (2002). Xenotransplant cardiac chimera: immune tolerance of adult stem cells. Ann Thorac Surg.

[38] MacDonald DJ, Luo J, Saito T, Duong M, Bernier PL, Chiu RC, Shum-Tim D (2005). Persistence of marrow stromal cells implanted into acutely infarcted myocardium: observations in a xenotransplant model. J Thorac Cardiovasc Surg.

[39] Le Blanc K, Mougiakakos D (2012). Multipotent mesenchymal stromal cells and the innate immune system. Nat Rev Immunol.

[40] Bassi EJ, Mayora Aita CA, Saraiva Câmara NO (2011). Immune regulatory properties of multipotent mesenchymal stromal cells: where do we stand?. World J Stem Cells.

[41] Nauta AJ, Fibbe WE (2007). Immunomodulatory properties of mesenchymal stromal cells. Blood..

[42] Grinnemo KH, Månsson A, Dellgren G, Klingberg D, Wardell E, Drvota V, Tammik C, Holgersson J, Ringdén O, Sylvén C, Le Blanc K (2004). Xenoreactivity and engraftment of human mesenchymal stem cells transplanted into infarcted rat myocardium. J Thorac Cardiovasc Surg.

